# Seeing Is Believing: Nuclear Imaging of HIV Persistence

**DOI:** 10.3389/fimmu.2019.02077

**Published:** 2019-09-12

**Authors:** Timothy J. Henrich, Priscilla Y. Hsue, Henry VanBrocklin

**Affiliations:** ^1^Division of Experimental Medicine, Department of Medicine, University of San Francisco, San Francisco, CA, United States; ^2^Division of Cardiology, Department of Medicine, University of San Francisco, San Francisco, CA, United States; ^3^Radiopharmaceutical Research Program, Center for Molecular and Functional Imaging, University of San Francisco, San Francisco, CA, United States

**Keywords:** human immunodeficiency virus, positron emission tomography imaging, simian immunodeficiency virus, nuclear medicine, molecular imaging

## Abstract

A major obstacle to HIV eradication is the presence of infected cells that persist despite suppressive antiretroviral therapy (ART). HIV largely resides outside of the peripheral circulation, and thus, numerous anatomical and lymphoid compartments that have the capacity to harbor HIV are inaccessible to routine sampling. As a result, there is a limited understanding of the tissue burden of HIV infection or anatomical distribution of HIV transcriptional and translational activity. Novel, non-invasive, *in vivo* methods are urgently needed to address this fundamental gap in knowledge. In this review, we discuss past and current nuclear imaging approaches that have been applied to HIV infection with an emphasis on current strategies to implement positron emission tomography (PET)-based imaging to directly visualize and characterize whole-body HIV burden. These imaging approaches have various limitations, such as the potential for limited PET sensitivity and specificity in the setting of ART suppression or low viral burden. However, recent advances in high-sensitivity, total-body PET imaging platforms and development of new radiotracer technologies that may enhance anatomical penetration of target-specific tracer molecules are discussed. Potential strategies to image non-viral markers of HIV tissue burden or focal immune perturbation are also addressed. Overall, emerging nuclear imaging techniques and platforms may play an important role in the development of novel therapeutic and HIV reservoir eradication strategies.

## Introduction

Despite the overwhelming success of antiretroviral therapy (ART) to achieve complete or near-complete HIV suppression, residual virus that integrates into host cell genomes prior to ART initiation persists indefinitely. Blood-derived resting CD4+ T cells comprise one of the most characterized reservoirs of latent HIV, and integrated viral DNA can exist at frequencies below one copy per million resting CD4+ T cells (1–6). However, HIV largely resides in organized lymphoid or other tissues outside of the peripheral circulation, and many anatomical regions are inaccessible to routine sampling (7–16). Only a small amount of tissue from a small number of sites can be realistically obtained from living human participants, and one of the major barriers to the successful design and implementation of HIV eradication or immune-based therapeutic strategies is the limited ability to characterize the tissue-wide burden of HIV in the setting of ART.

HIV-1 infection leads to immune activation and inflammation throughout all stages of disease. Markers of T-cell activation remain elevated in blood and lymphoid tissues in HIV-infected individuals, even in the setting of elite control or after years of suppressive ART. Certain immune privileged environments may be especially important foci of HIV persistence and viral transcriptional activity. For example, CD4+ T-follicular cells (T_FH_) within lymph node B cell follicles have been shown to be highly enriched in HIV-1 DNA, are very permissible to HIV infection, and are able to produce high levels of replication competent virus upon *ex vivo* stimulation (12, 17–19). T_FH_ cells may be protected from various host immune responses by their location in the unique histological makeup (12, 17–19). Even outside of infected tissues, persistent HIV has lasting and often profound effects on tissues such as vascular endothelium, gut, and brain, and leads to sustained, systemic inflammatory responses. Markers of inflammation, coagulation, and immune activation remain elevated in effectively treated HIV infection and are strong predictors of mortality and non-AIDS events, which has been demonstrated in a variety of cohorts (20–23). As a result, there are direct and indirect consequences of HIV infection that are clinically relevant, even in the setting of treated and suppressed HIV. For example, HIV has been associated with increased cardiovascular disease, neurological disorders, and various hematological and solid-tumor malignancies (24).

The direct and indirect impact of persistent HIV on immune activation, systemic inflammation, and increased clinical comorbidities has led to interest in positron emission tomography (PET) and other molecular imaging techniques as tools to better understand the whole-body burden and consequences of HIV infection. Molecular imaging has been critical for the diagnosis, treatment, and management of various malignancies and other diseases. Similar modalities have the potential to provide insights into the design, implementation, and analysis of immunotherapies and other interventions to reduce HIV reservoir burden, lower inflammation, and thus reduce HIV-related morbidity.

## Nuclear Imaging Approaches to HIV Persistence and HIV-Related Morbidity

### The Molecular Imaging Toolbox

Innovative strategies to perform molecular imaging, from microscopic visualization and characterization techniques on the tissue level, to whole-body *in vivo* anatomical and functional imaging incorporating techniques such as SPECT and PET, are rapidly being developed for a wide range of diseases, including HIV and other chronic infections (see Table 1).

**Table 1:** Historical and current PET radiotracers used in the context of HIV infection.

**Early SPECT radiotracer**	**Target or response in disease**
^99m^Tc-HMPAO	Cerebral blood flow (25–34)
^123^I-Iodoamphetamine	Cerebral blood flow (35–38)
^123^I-FP-CIT	Cocaine analog, dopaminergic neurotransmission (39)
^123^I-iodobenzamide	Dopaminergic neurotransmission
^201^Thallium	Differentiation of CNS lymphoma from toxoplasmosis (40–43)
**Current (dates) PET radiotracers**	**Target or response in disease**
^18^F-Fluorodeoxyglucose FDG	Glucose metabolism
TSPO imaging (^11^C-PBR28, ^18^F-DPA-714, ^11^C-DPA-713, ^11^C-PK11195)	Neuroinflammation (44–50)
Fluoromisonidazole	Reduced hypoxia associated with Nelfinavir (51)
^82^Rb	Myocardial perfusion (52, 53)
^11^C-DASB	Dysregulated serotonergic transmission (54, 55)
^11^C-PiB	Alzheimer disease (AD) plaque tracer—no increased AD risk (56, 57)

*Ex vivo* molecular imaging on the cellular and tissue level has already provided many important insights into HIV pathogenesis such as identifying foci of residual infected cells in the setting of ART and characterizing the immunological microenvironments of such foci (58–65). These studies have focused largely on gut, lymphoid, and central nervous system tissues but may involve a wide variety of other scenarios such as tumor microenvironments and quantifying vascular inflammation. However, the focus of this review covers *in vivo* nuclear medicine approaches with an emphasis on novel PET imaging approaches of HIV persistence.

### Nuclear Imaging Approaches to HIV Infection

Common nuclear imaging approaches that have been applied to HIV infection for over 20 years include SPECT/CT and PET/CT imaging (44). These modalities involve the detection, anatomical location, and kinetics of radioactive tracer uptake, with SPECT involving the detection of single photon gamma emission and PET measuring positron emission. Clinically, these nuclear imaging modalities are commonly used to diagnose various malignancies and provide information on potential tumor burden or sites of metastases, disease staging, and response to various treatment strategies. They are also used to differentiate benign, metabolically quiescent tissues from metabolically active foci, which may be manifested by active infections, reactive lymphoid tissues, vascular inflammation, and more. As a result, nuclear imaging has been applied in the setting of HIV infection and HIV-related comorbidities. HIV imaging studies are diverse and have involved numerous tracers and measured outcomes. As summarized in Table 1 and below, PET imaging has been used to (1) measure cellular metabolic activity in a variety of different clinical scenarios (e.g., 18F-FDG); (2) carry out anatomical and functional neuroimaging involving various metabolic measures, cerebral fluid, dopamine transport, and cellular activation in the setting of HIV-associated neurological disease (HAND), central nervous system malignancies, and opportunistic infections; (3) determine ART-related toxicities; (4) quantify changes in various immune cell types, such as CD4+ T-cell distribution in the setting of immunomodulatory therapies in animal studies; and (5) characterize the effects of HIV on cardiovascular disease. A recent PubMed search using HIV or AIDS and PET yielded 537 references, averaging about 10 articles per year.

Over the past several years, there has been increased interest in the development of HIV-specific tracers to provide direct anatomical localization and burden of infection. *In vivo* studies are currently taking place using techniques such as radiolabeling monoclonal antibodies (mAbs) specific for HIV or SIV envelope proteins (66, 67). In addition, traditional nuclear medicine approaches, such as FDG-PET, have been applied to look at HIV persistence in the setting of active infection, HIV controllers (i.e., those who are able to suppress virus without ART), and ART-suppressed individuals (see discussion below). These immunoimaging approaches have the potential to significantly improve our understanding of where and how residual viral replication and HIV-related inflammation resides in the setting of suppressive therapy. More specifically, the diverse nuclear imaging toolbox may prove to be useful in people living with HIV to:

- Understand the temporal changes that occur within the whole body as a function of disease status, ART use, viral recrudescence following cessation of therapy, or foci of HIV reactivation during a “shock and kill” approach to HIV remission.
- Distinguish opportunistic infections and malignancies from direct or indirect impact of active or suppressed HIV infection.
- Assist in the development of new drugs and therapeutic paradigms.
- Aid in participant selection for various therapeutic strategies.
- Monitor individualized responses to various therapeutic interventions (including ART, immunotherapies, etc.).

### Radiopharmaceutical, Pharmacokinetic, and Nuclear Imaging Considerations

The utility of a specific nuclear imaging strategy is tightly linked with the various properties of the applied radiopharmaceutical tracer. These properties include radiologic dose, exposure, decay rates and tissue uptake, drug metabolism, and excretion. PET tracers involve a radiolabeled molecule as a source of positrons. These isotopes have a wide range of radiological half-lives (*t*_1/2_). Decay rates range widely from minutes to many days as summarized in Table 2, and ideally are in synergy with the pharmacokinetics of the radiolabeled tracer. For example, mAbs may take several days to reach target tissues and bind to specific targets, therefore requiring longer-acting isotopes such as zirconium-89 (*t*_1/2_ = 78 h), whereas FDG uptake (fluorine-18 *t*_1/2_ = 110 min) is rapid and glucose is internalized relatively quickly by metabolically active cells. Special care in matching the appropriate radioactive molecule with the target drug will be critical in the rational design and implementation of HIV-specific imaging agents. In addition, human studies are limited by the total radiation exposure to a participant, leading to challenges with administration of high enough doses for clinically meaningful target-to-background contrast, restricting the frequency of tracer administration and may limit longitudinal imaging studies. In addition, target densities may be quite low in various clinical scenarios such as ART-suppressed HIV infection, where viral proteins may be expressed in very low amounts or frequencies on cells or in tissue, if at all. As a result, there are expected to be significant challenges to increase signal-to-noise ratios in these participants, and this highlights the continued need for non-viral-specific tracers to provide information on location, burden, and immunological impact of persistent HIV infection.

**Table 2:** Common radioisotopes used in HIV nuclear imaging.

**Radioisotope**	**Half-lives**	**Pros and cons**
^11^C	20 min	Short half-life good for repeat studies, carbon-11 for carbon-12 exchange in small molecules/drugs produce the same labeled molecule/drug, half-life may be too short to achieve adequate signal-to-noise ratio, may not be transported to distant scanners
^18^F	110 min	Ideal positron emission characteristics for high-resolution PET imaging may incorporate into small-molecules/drugs. Half-life suitable for longer imaging and delivery to remote scanner sites. May not be long enough for larger biologic molecules. Free ^18^F-Fluoride ion accumulates in bone
^64^Cu	12.7 h	Half-life compatible with imaging larger molecules like mAbs. However, half-life may limit utility when using HIV gp120-specific or other mAb, which take time to penetrate certain target tissues
^89^Zr	78 h	Half-life compatible with imaging larger molecules like mAbs. Radiation dose to patient is higher so lower administered dose is necessary. Takes a long time to clear from body so repeat studies limited but allows for serial imaging over days with a single radioisotype injection. May be beneficial when using HIV gp120-specific mAb, which takes time to penetrate certain target tissues. Ideal for transport to distant scanners. ^89^ZrCl_3_ may accumulate in active bone

### PET Imaging in HIV Infection—Cellular Metabolic Activity, Immune Activation, and HIV Persistence

In the research setting, PET/CT has commonly been used in conjunction with FDG, which provides a measurement of glucose metabolism as a surrogate for inflammation, which is taken up substantially higher by inflammatory cells and macrophages in the tissue (68, 69). FDG-PET imaging has been reported for HIV in the mid to late 1980s, with monitoring of HIV pre- and post-AZT monotherapy (combination ART was not widely available until the mid-1990s), and workup of HIV-associated neurological disorders along with staging of malignancies (44, 70, 71). In addition, FDG-PET studies have involved anatomical localization of HIV-associated immune activation, correlating lymph node inflammation with disease stage, and associating high areas of FDG uptake in non-human primates with productive SIV infection (72–77). Since this time, studies in the general population have demonstrated that arterial inflammation assessed using FDG-PET/CT can predict future cardiovascular (CV) events (78). Furthermore, lipid lowering using statin therapy along with thiazolidinedione therapy has reduced arterial FDG-PET uptake in several clinical trials (79–83). Our group also has recently reported that using a mAb to IL-1β significantly reduced inflammatory markers along with arterial and bone marrow metabolic activity assessed using FDG-PET/CT in the setting of treated HIV (84). Studies involving animal models and humans showed that both relative and absolute FDG uptake within inflamed tissues (e.g., atherosclerotic plaques) correlate with the degree of immune cell infiltration (12, 17–19, 85–89). More recently, FDG-PET has been applied to assess altered glucose metabolism in HIV-associated inflammation and has demonstrated that HIV patients have higher arterial inflammation that is associated with sCD163 (87). Initiation of ART reduced bone marrow activity but did not affect arterial inflammation; furthermore, metabolic activity on FDG-PET/CT prior to ART was predictive of immune reconstitution inflammatory syndrome development (90).

Subsequently, our group showed that HIV-infected individuals on ART have higher metabolic activity as measured by FDG-PET/CT in the arterial vasculature and lymph nodes than matched uninfected controls and that these markers correlated with measures of HIV persistence in peripheral blood (91). Importantly, individuals on ART had higher FDG uptake in lymph nodes and arterial vasculature than matched uninfected controls. Overall, lymph node FDG activity was significantly associated with levels of integrated HIV DNA measured in peripheral blood mononuclear cells (91). This study suggests that PET-based imaging of inflammation or immune activation has the potential to provide information regarding regional areas of HIV persistence. However, FDG is likely taken up by immune activation/inflammation even when not in tissue with HIV-persistent foci (e.g., arterial wall, which may be influenced by monocyte activation); therefore, more specific markers of T-cell trafficking and targeting of infected tissues are needed.

Recently, advances in molecular imaging of immune activation by PET have made it possible to use non-invasive strategies to monitor immune activation with increased T-cell specificity than FDG. Increased activity of nucleoside salvage pathways has been associated with the proliferation of adaptive immune cells (92). In preclinical models, the PET probe [^18^F]-2-fluoro-d-(arabinofuranosyl)cytosine ([^18^F]-FAC), which targets the deoxycytidine salvage pathway, was shown to localize to focal sites of immune activation (93) and is predominantly accumulated in proliferative T cells (94). Recently, a radiofluorinated imaging agent [^18^F]F-AraG (95) was synthesized with a goal of development for human use. F-AraG is a fluorinated purine derivative with selective T-cell uptake. A water-soluble AraG prodrug, Nelarabine, is FDA-approved for the treatment of relapsed T-cell acute lymphoblastic leukemia and T-cell lymphoblastic lymphomas (96, 97). [^18^F]F-AraG is a high-affinity substrate for deoxyguanosine kinase (dGK) and a low-affinity substrate for deoxycytidine kinase (dCK). Both dGK and dCK are over-expressed in activated T cells. Blocking the expression of either dGK or dCK causes reduction in [^18^F]F-AraG uptake, while over-expression of either dGK or dCK leads to increased accumulation of [^18^F]F-AraG. T-cell-specific tracers such as these may play an important role in imaging HIV persistence, with the potential to be more specific to regional areas of immune perturbance as a result of HIV replication or residual viral transcriptional activity.

### Neuroimaging Microglia Activation in HIV Infection and Related Neurologic Disorders

PET imaging using tracers specific for activated microglial cells is another example of how non-specific markers of increased immune activation has been successfully applied to study HIV-related comorbidities in the central nervous system. More specifically, molecules have been developed that target the 18-kDa mitochondrial translocator protein (TSPO) that shuttles cholesterol into mitochondria for steroid biosynthesis (45–50). TSPO is upregulated in activated microglia, and, as a result, has been used in neuroimaging to determine differences between HIV-infected and uninfected individuals and to characterize differences between various HIV clinical disease manifestations, including HAND (50). PET imaging with TSPO-specific tracers appear to be more specific to innate immune activation than FDG (45) and have led to some important insights into central nervous system persistence of HIV. For example, ART-suppressed individuals without cognitive impairment have been observed to have chronically elevated microglial activation (48), whereas other studies showed that TSPO levels correlated with worse executive brain performance and other HIV-associated cognitive vulnerabilities (46, 49). Despite varying results complicated by various experimental designs and definitions of cognitive impairment (50), there is continued interest in using PET-based immune activation approaches to study the direct impact of residual HIV infection in the setting of suppressive ART.

### Antiretroviral Drug Labeling

The question of whether or not there is ongoing replication in various tissue sanctuaries in the setting of otherwise suppressive ART remains controversial. For example, there is a paucity of robust phylogenic evidence for evolution of HIV sequences or development of resistant mutations in suppressed individuals over time and ART intensification studies have not demonstrated reductions in low-level, residual plasma HIV RNA levels (98–103). Many of these studies were performed in peripheral blood or limited by the depth of sequence coverage or tissues sampled. Other studies have shown potential indirect evidence of replication such as an increase in unintegrated episomal HIV DNA in blood and cell-associated RNA in tissue (104–106). One topic of interest is the extent to which various ART drugs reach or have activity in various anatomical tissue compartments (107), potentially creating viral sanctuaries that permit low-level replication or, at the very least, allow higher levels of viral transcriptional activity (9, 106, 108). Transcriptionally active cells may also lead to chronic immune activation and inflammation. However, sampling all of the potential sites of persistent HIV for concomitant ART concentrations and viral reservoir persistence is not practical. It is also difficult to obtain information on the kinetics of drug distribution within tissues outside of peripheral blood. As a result, PET-based imaging of radiolabeled antiretroviral drugs may play an important role in pinpointing areas of poor ART penetration and therefore important sites of persistent HIV burden and potential foci of viral rebound following ART cessation. Imaging studies using fluorine-18-labeled raltegravir (a strand-transfer integrase inhibitor) are ongoing (NCT03174977) and have the potential to locate areas of HIV persistence.

### PET Immunoimaging of CD4+ T Cell Dynamics in SIV Infection

CD4+ T cells are the main target of HIV infection. Active disease leads to subsequent and profound reduction in CD4+ lymphocytes throughout the blood and tissues. While counts may improve in many individuals on ART, lasting perturbations to tissues such as the lymph nodes and gut-associated lymphoid tissues are common (8, 109–114). As a result, there has been interest in CD4+ T-cell-specific PET-based imaging techniques to follow CD4+ T-cell dynamics and recovery following various interventions. A recent investigation of the use of an α4β7 mAb in acute SIV infection in macaques demonstrated sustained virological control in mAb-treated monkeys. While these results have yet to be confirmed, the study involved PET-CT imaging using a ^64^Cu-labeled F(ab′)2 antibody against CD4. The study demonstrated repopulation of CD4+ T cells in a number of tissues, including gut, which was unexpected based on the original study hypothesis that the α4β7 mAb would interfere with CD4+ T-cell trafficking to these areas (67). This investigation is an example of how imaging various cell-specific markers may provide critical information regarding whole-body responses to various immune-based or other therapies for a wide variety of diseases. For example, CD8+ T-cell responses can theoretically be tracked over time in response to interventions such as vaccines or therapies that remove immune checkpoint and reverse T-cell exhaustion (e.g., anti-PD1 therapy).

### PET-Based Direct Imaging of SIV Infection

As above, PET-based imaging techniques have the potential to delineate tissue burden and sequelae of HIV infection. PET/CT imaging approaches using a radiolabeled ^64^Cu-labeled SIV gp120 mAb-specific clone (7D3) have been recently applied to assess SIV envelope protein expression in infected macaques with varying degrees of viremic control and in the setting of early initiation of ART (66). Results from this pivotal study demonstrated that areas of active SIV replication can be visualized and distinguished from non-selective tracer uptake in uninfected animals, with some HIV-related signal detected several weeks following ART initiation. As would be expected, lymphoid-rich areas were localized predominately at sites of persistent SIV protein expression (66). The study also showed that anatomical regions that are often neglected by *in vivo* tissue sampling, such as nasal-associated lymph node tissue, may play an important role in initial HIV seeding and subsequent persistence. A follow-up sub-study of anti-α4β7 treatment in SIV-infected macaques incorporating the radiolabeled SIV gp120 mAb demonstrated a reduction in SIV protein expression in various tissues, including the lung, spleen, and lymph node chains (89). These data suggest that direct SIV or HIV imaging radiotracers have the potential to play a critical role in characterizing HIV persistence and response to curative strategies. As a result, there is currently a high level of interest in direct HIV imaging techniques to humans. However, immunoimaging in SIV infection does have several potential limitations. For example, mAb or antigen binding fragments may have heterogeneous tissue distribution *in vivo*, and humanization or simianization may lead to immunogenicity concerns (115). Finally, the SIV or HIV antigen-specific PET-imaging approaches do not allow for direct discrimination between actively viral producing cells, cells expressing SIV or HIV antigens at the surface, viral particles, or simply viral antigen trapping by non-infected cells.

### Human HIV-Specific PET Imaging: Challenges and Promises

Despite the early success of direct SIV specific in the first non-human primate PET/CT imaging studies, there are several challenges in adopting these techniques to human imaging. For example:

1. Non-human primates are typically infected with a clonal SIV strain with known binding affinity to gp120-specific mAb. HIV-infected humans can be extraordinarily diverse with both minority and majority clones capable of harboring resistance mutations to the clinically available HIV-specific mAbs, which have been previously developed as therapeutic broadly neutralizing antibodies (116–122). As a result, there is expected to be a wide range of mAb binding affinities between study participants that will require implementation of mAb resistance testing and careful considerations as to data analysis and interpretation.
2. HIV gp120 expression is expected to be very low among infected tissues in participants on suppressive ART. As a result, there may be insufficient signal-to-noise ratio in order to visualize areas of persistent infection. However, PET imaging may be particularly useful during early infection and for characterizing foci of early tissue HIV recrudescence following cessation of ART; incorporating PET imaging approaches in studies involving analytical ART interruptions is of utmost importance.
3. mAbs do not readily cross the blood–brain barrier. Barring any inflammation and major perturbations of the blood–brain barrier, imaging potential foci of HIV in the central nervous system will be challenging. As a result, the development of small-molecule HIV-specific tracers with improved central nervous system or other immune privileged tissue penetration is urgently needed.
4. Longitudinal human trials are limited by radiation exposure; therefore, multiple imaging time points may be difficult to incorporate into a variety of studies. This may be a particular issue when implementing tracers conjugated with radioisotopes with longer half-lives *in vivo*, which are likely going to be required given the kinetics of mAb uptake as discussed above. These limitations provide the rationale to incorporate more than one radiotracer in human studies. For example, administering an HIV-specific mAb tracer following PET imaging using a non-viral specific marker of inflammation or immune activation may provide important insights into the relationship between ongoing immune perturbations and HIV persistence.

Fortunately, several strategies exist or are in development to address these challenges using radiolabeled mAbs in PET imaging. For example, smaller affibody proteins or antibody fragments (e.g., minibodies, nanobodies, and single-chain variable regions) (123–125) may have improved tissue penetration and favorable pharmacokinetics for imaging low-level HIV protein expression in various tissues. There is also a high level of interest in the development of dual or multi-targeted molecules for immunoimaging (126) or engineering antibodies to have greater anatomical barrier penetration. One exciting strategy is increasing antibody delivery across the blood–brain barrier by developing bispecific antibodies or designer molecular shuttles that bind to the transferrin receptor (127–130). Animal studies are exciting and can theoretically be applied to HIV-specific mAb or antibody fragments.

The development and implementation of very-high-sensitivity, total-body PET scanners, such as the EXPLORER platform (131–133), are also likely to overcome some of the signal-to-noise limitations of imaging HIV-infected cells in ART-suppressed individuals or those with low overall HIV envelope protein expression. These platforms are just now coming on line for *in vivo* use, and have the potential to revolutionize immunoPET imaging. Approximately 1% of the photons emitted during traditional PET scanning are detected given a limited axial field of view and body length that can be imaged at one time. The field of view in EXPLORER is extended to the entire individual by using a large number of parallel detectors that simultaneously detect photon emission (134). Early data suggest that EXPLORER PET provides a >40-fold gain in effective sensitivity and a >6-fold increase in signal-to-noise ratio compared with standard PET scanners (135). The first-in-human imaging studies have recently been completed (131) and offer an opportunity to significantly advance PET-based imaging of HIV reservoirs. Other emerging technologies include solid-state digital photon counting PET systems, such as those that use solid-state silicon photomultiplier technology (136). These systems have led to improvements in signal-to-noise ratios and enhancing image contrast (137, 138) and may play an important role in improving PET imaging in HIV infection.

### Limitations of *in vitro* Modeling of HIV-Specific Immunoimaging Techniques

HIV or SIV envelope-specific PET immunoimaging strategies are likely to be semiquantitative at best. For example, PET/MR or PET/CT imaging techniques reveal relative changes in mAb tracer uptake in various tissue region of interest (e.g., lymph node tissues, gut) before or after initiation of ART or immunotherapy (66, 67). However, questions arise as to what the intensity of the PET signal means in terms of the actual number of infected, HIV or SIV envelope-expressing cells. In other words, can PET imaging be used to directly quantify the burden of HIV *in vivo*? One solution that is often presented is to perform *ex vivo* studies involving PET imaging of three-dimensional clusters of known numbers of infected and uninfected cells (either laboratory infected or derived directly from infected individuals) in order to determine the sensitivity of PET to detect various levels of HIV protein expression. While appealing, these studies are limited by the multitude of variables within living organisms that determine tracer uptake and PET detection. Modern PET scanners are sensitive and able to detect tracer-derived positron emission events above normal background radiation (139). Simply labeling a cell or a group of cells that express HIV envelope will likely lead to a detectable signal. However, regardless of what threshold in the number of infected cells can be detected (e.g., 10, 100, or 1,000 in a sub-centimeter cluster) in isolation, these types of *ex vivo* experiments are unable to account for many biasing factors. For example, radiotracers are often delivered in microdoses, with or without a specified amount of unlabeled antibody. The distribution of these microdoses to various tissues relies on many variables, such as blood flow dynamics, tissue fibrosis, and non-specific tracer uptake, to name just a few. In addition, there is background radiation that is given off by tracers in the macro and microcirculation and from organs involved in tracer metabolism and excretion. Coupled with the need for PET attenuation and tomographic reconstructions in image acquisition and analysis, it will likely be difficult to correlate readout of *ex vivo* PET sensitivity studies with actual uptake in living organisms. In addition, each individual has different metabolic and physiologic dynamics (e.g., liver function, cardiac output, body surface area and mass, renal glomerular filtration rates, local microanatomical variations, etc.). As a result, performing parallel *in vivo* tissue biopsy studies along with PET imaging may be the most useful strategy to provide some quantitative understanding of radiotracer uptake signal and direct cellular measures of HIV burden or cell activation state.

## Conclusions

PET imaging offers several exciting strategies to characterize HIV and HIV-related comorbidities. Despite limitations of traditional of nuclear imaging techniques in identifying HIV-infected cells *in vivo*, proof-of-concept SIV non-human primate studies demonstrate that various immunoimaging approaches have potential to enhance HIV curative and persistence research. Signal-to-noise issues are likely to limit imaging in ART-suppressed individuals when cell-surface HIV protein expression is expected to be low. However, novel approaches such as high-sensitivity, total-body EXPLORER imaging, PET imaging during latent HIV reservoir reactivation or ATI, and development and implementation of non-viral markers of HIV persistence have the capacity to overcome these limitations and provide important tools for the development of novel therapeutic strategies. In addition, technical and data processing advancements may allow for combination imaging approaches, from tissue-level microscopy to whole-body PET imaging.

## Author Contributions

TH, PH, and HV wrote the manuscript and obtained funding.

### Conflict of Interest Statement

The authors declare that they received funding from Merck for a study discussed in this review. The funder provided grant support to perform PET-MR imaging of raltegravir. PH received honoraria unrelated to the topic of the paper from Gilead and Merck. TH has received honoraria unrelated to the topic of the paper from Merck. The remaining author declares that the research was conducted in the absence of any commercial or financial relationships that could be construed as a potential conflict of interest.
